# T cell receptor repertoire characteristics and therapeutic potential of tumor infiltrating lymphocytes (TILs) derived from metastatic lymph node in cervical cancer

**DOI:** 10.1186/s43556-024-00215-w

**Published:** 2024-11-04

**Authors:** Xuan Zhao, Zhenjiang Liu, Haifeng Qin, Yarong Liu, Yi Zhang

**Affiliations:** 1 Biotherapy Center and Cancer Center, The First Affiliated Hospital of Zhengzhou University, Zhengzhou, Henan 450052 China; 2 Grit Biotechnology Ltd, Building No. 24, 388 Sheng Rong Road, Pudong District, Shanghai, 201210 China; 3 Department of Oncology, Chinese PLA General Hospital, No. 8 of Dongda Street, Fengtai District, Beijing, 100071 China

## Dear Editor,

Cervical cancer, a leading reproductive malignancy, sees over 570,000 new cases and 311,000 deaths annually, predominantly in low- and middle-income countries. Conventional chemotherapy offers limited efficacy, with an overall response rate (ORR) as low as 13% and median progression free survival (PFS) and overall survival (OS) ranging from 2.8 to 8 and 6–19 months in advanced cervical cancer, respectively. Immunotherapy, particularly anti-PD-1 mAb together with anti-CTLA-4 mAb, shows promise with an ORR of 36% [1]. Adoptive cell therapy by tumor-infiltrating lymphocytes (TILs) achieved a 44.4% ORR in advanced cervical cancer [2]. At this point, we report clinical findings of one patient with recurrent cervical cancer and multiple metastasis treated with autologous TILs therapy (GT101) together with IL-2 injection after sequential modified lymphodepleting regimen. Partial response (PR) after TILs treatment was observed with limited and tolerable immune-related adverse event (irAE). Subsequent TCR repertoire sequencing in peripheral blood demonstrated robust expansion of dominant clones from TILs product and long-term persistence in vivo.

**Fig. 1:**
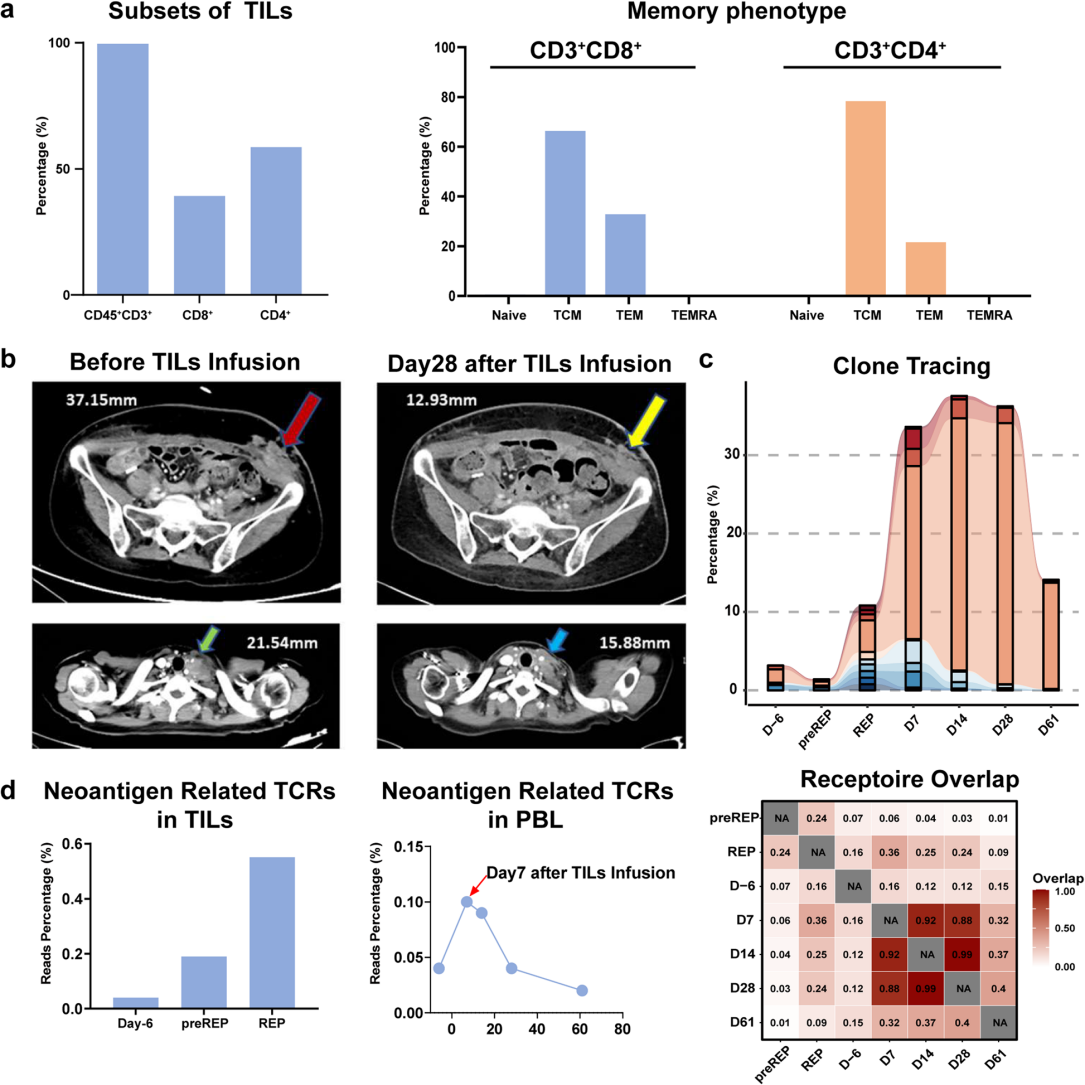
Phenotypic profiling of TILs product, imaging-based assessment of therapeutic efficacy and longitudinal monitoring of TIL persistence post-infusion in vivo. (**a**) Left panel: Percentage of CD3^+^, CD4^+^ and CD8^+^ T cells in final product of TILs. Right panel: Memory phenotype of TILs product. (**b**) Computed tomography scans showed subcutaneous metastasis of left lower abdomen (upper left panel, red arrow) and the supraclavicular nodes of the left side (lower left panel, green arrow) at baseline before TILs therapy with IL-2 as adjuvant. Computed tomography scans obtained in January 10th 2022 (28 days after TILs infusion) showed that shrinkage of the supraclavicular nodes of (upper right panel, yellow arrow) coincident with the left side the subcutaneous metastasis of left lower abdomen (lower right panel, blue arrow). (**c**) Top panel: TCR of peripheral blood T cells clone tracking by TCRβ chain sequencing. Bottom panel: TCR repertoire similarity analysis between TILs product and T cells derived from peripheral blood before and after infusion. (**d**) Left panel: Percentage of putative neo-antigen related TCR in TILs product at indicated time points and Day-6 before TILs infusion. Right panel: Percentage of putative neo-antigen related TCR in peripheral blood T cells at indicated time points

TILs from a patient with advanced cervical cancer were successfully expanded from metastatic inguinal lymph nodes. At the end of rapid expansion phase (REP), TILs product was harvest as almost absolute CD45^+^CD3^+^T cells (99.6%) with 58.6% CD4^+^ and 39.2% CD8^+^ T cell subsets, respectively (Fig. 1a Left panel). TILs products exhibited the relatively dominant central memory T-cell phenotype (CD45RO^+^CD62L^+^) both in CD4^+^ T cells (78.3%) and CD8^+^ T cells (66.4%), which might be positively related with its strong proliferative potential in patient and provide long-term antitumor protection (Fig. 1a Right panel).

The patient was treated with autologous TILs therapy (GT101) and IL-2 injection following a modified lymphodepleting regimen. After a non-myeloablative lymphodepleting regimen consisting of cyclophosphamide (300 mg/m^2^ for 3 days) and fludarabine (25 mg/m^2^ for 3 days), the patient was infused with 1.5 × 10^10^ autologous TILs (GT101) followed by just two doses IL-2 administration (1.8 × 10^7^ IU at day 1 and 0.9 × 10^7^ IU at day 2). Both metastatic lesions had regressed at the time point of first assessment, namely 28 days after TILs infusion. The efficacy was evaluated as Partial Response (PR) at the first assessment according to the Response Evaluation Criteria in Solid Tumors (RECIST) v1.1 (Fig. 1b). The patient experienced fever together with chills, hypotension, syncope and diarrhea. The adverse event profile was generally consistent with lymphodepletion and IL-2 regimens as well as the underlying advanced disease.

To evaluate the evolution dynamics of TILs after infusion, counts of major immune subsets and cytokine level in peripheral blood were monitored at a series of time points including 1 and 6 days before TILs treatment and day 1, 3, 7, 14, 28, 61 and 70 days after infusion, suggesting good engraftment and expansion of TILs post infusion (Fig. Sa-f).

To track the expansion of TCR clones in infused TILs product, we performed quantitative analysis of T cell receptor β (TRB) clonotypes according to the 5’ Rapid Amplification of cDNA Ends (5’ RACE) protocol. The frequency of the top10 clonotypes in REP product was tracked and highlighted among samples of PBMCs and preREP TILs samples at indicated time points (Fig. 1c Top panel). These top TCR clones in REP experienced nearly three-fold expansion in 7 days after infusion, which could still be identified even after two months, especially the top 1 TCR that dominantly existed at most of time points. To further assess the TCR repertoire similarity among detected TCRs, we performed Morisita-Horn (MH) similarity analysis of TRB CDR3. Compared with PBMCs sample from D-6 (6 days before infusion), REP TILs showed higher TCR repertoire similarity with post-infusion PBMC samples (Fig. 1c Bottom panel). The similarity between REP and PBMC samples on D7, D14 and D28 is higher than 0.20, while the similarity between D-6 and D7, D14 and D28 is lower than 0.20, indicating the infusion induced remarkable change of the patient’s TCR clonotypes in peripheral blood. The top 10 TCRs in PBMC at D7 could all be traced in REP sample, and most of them (9 out of 10) were dominant clones in REP ranked within top 100.

A memory-progenitor stem-like phenotype, scilicet CD39^−^CD69^−^CD8^+^ subset, has been reported to be associated with therapeutic benefits due to their capacity of self-renewal, expansion, persistence and superior antitumor response in patients [3]. Hence, certain percentage of CD39^−^CD69^−^ in CD8^+^ TILs (up to 33.9%) might be associated with its substantial expansion and subsequent antitumor efficacy in the patient. In order to predict the putative neoantigen reactive TCR (Neo-TCR), McPas database with a catalogue of TCR sequences linked to their tumor antigen target was used to annotate Neo-TCR by TRB CDR3 sequence. Clonotypes with the same CDR3 amino acid sequence as the Neo-TCR records in the database were defined as Neo-TCR. The percentage of the putative Neo-TCRs was elevated in preREP TILs than baseline PBMC (D-6), and the REP manufacture process further promoted the enrichment of these Neo-TCRs in the final product (Fig. 1d Left panel). In the patient’s peripheral blood, the Neo-TCR ratios in D7 and D14 are higher than D-6, which may indicate that the Neo-TCRs derived from the TILs product engrafted and/or expanded post infusion and gradually declined at later time points (Fig. 1d Right panel).

HPV antigen specificity of TOP1-6 TCRs in Day 7 peripheral blood after infusion was tested as the patient was HPV16 infected. Peripheral T cells from a healthy donor were transduced with TOP1 TCR as experimental group and a published HPV-specific TCR (HPV TCR-T) as the positive control. Two MHC partly matched HPV-positive tumor cell lines, CASKI and SIHA, were used as target cells. Post 48 h co-incubation with TCR-T cells at varying E/T ratios, the TOP1 TCR showed no killing effect to target cells in comparison with the strong killing function of HPV-specific TCR-T (Fig. Sg). Next, Jurkat-NFAT-Luciferase reporter cells were transduced with the TOP 2–6 TCRs (Fig. Sh), but no antigen-induced luciferase signal was observed upon co-culture with CASKI at different E/T ratios except the HPV-specific TCR, suggesting non-reactivity to HPV antigens (Fig. Si). Based on the previous study on single-cell sequencing analysis and the TCR tracking of TILs product and patients’ blood samples post TILs transfer, we remain convinced that the dominant TCR clones in peripheral blood post TILs infusion are closely associated with the patient’s clinical benefit [4, 5]. While the top clones in this case do not exhibit HPV reactivity as initially hypothesized, they are likely targeting neoantigens, tumor-associated antigens, non-coding region antigens, and/or cryptic antigens, which will be further investigated in future studies.

In this study, one-time treatment with GT101 TILs product and two dose IL-2 achieved PR in a post-chemotherapy and post-ICI treated patient with recurrent metastatic cervical carcinoma. Response was durable and AEs were transient and manageable. The TILs product was derived from the patient’s metastatic lymph nodes, which may be ideal sampling sites for TILs production due to the possible residence of T cells with good quality and/or high tumor specificity. Key attributes including quick expansion, a rational CD4/CD8 ratio, high percentage of memory T cells as well as the stem-like CD39^−^CD69^−^CD8^+^ subsets, may collectively contributed to the clinical efficacy in the patient. TCR tracing analysis showed highly proliferated and sustained TCR clones post TILs infusion in the peripheral blood with putative reactivity to neoantigens. These findings support the potential of TILs derived from metastatic lymph nodes as an effective therapeutic option for refractory cervical carcinoma patients.

## Supplementary Information

Supplementary Material 1.

## Data Availability

All data included in this study are available upon request by contact with corresponding author.
