# Supplementary material for: T cell receptor repertoire characteristics and therapeutic potential of tumor infiltrating lymphocytes (TILs) derived from metastatic lymph node in cervical cancer
Source: Mol Biomed. 2024 Nov 4;5:51. doi: 10.1186/s43556-024-00215-w (PMC11532323; doi:10.1186/s43556-024-00215-w)
Supplement: Supplementary file 1 — Supplementary Material 1. [file 43556_2024_215_MOESM1_ESM.zip › TILs in Cervival cancer Supplementary data.docx]

**Title page**

**T cell receptor repertoire characteristics and therapeutic potential of tumor infiltrating lymphocytes (TILs) derived from metastatic lymph node in cervical cancer**

Xuan Zhao^1*^, Zhenjiang Liu^2*^, Haifeng Qin^3*^, Yarong Liu^2+^, Yi Zhang^1+^

1. Biotherapy Center and Cancer Center, The First Affiliated Hospital of Zhengzhou University, Zhengzhou, Henan 450052, China
2. Grit Biotechnology Ltd., Building No. 24, 388 Sheng Rong Road, Pudong District, Shanghai, 201210, China

3. Department of Oncology, Chinese PLA General Hospital, No. 8 of Dongda Street, Fengtai, District, Beijing 100071, China

+: Correspondence: Yi Zhang [(yizhang@zzu.edu.cn)](mailto:(yizhang@zzu.edu.cn)), Yarong Liu ([yarong.liu@grit-bio.com](mailto:yarong.liu@grit-bio.com))

*: Xuan Zhao, Zhenjiang Liu and Haifeng Qin contribute equally to this work.

**Methods and Materials**

**TILs preparation**

Tumor tissues from the cervical cancer patients were processed for TIL expansion. The patient was eligible for tumor reduction surgery per NCCN guidelines. The study was approved by the IRB of The First Affiliated Hospital of Zhengzhou University and conducted per the Declaration of Helsinki.Primary tumors or lymph node metastases were chopped into 1–20 mm³ fragments and placed in a Grex-100 for pre-REP culture. In the REP phase, after 48 h stimulation with 30 ng/mL anti-CD3 antibody (ORTHOBIOTECH), feeders (irradiated PBMCs, 200x TILs) were added. TILs were maintained in T cell media with 3000 IU/mL rhIL-2 (T&L Biotechnology).

**Antibodies and flow cytometry**

Peripheral blood Samples were collected in patients and monitored for expansion of CD45^+^CD3^+^, CD3^-^CD19^+^, CD3^-^CD56^+^, CD3^-^CD14^+^, CD39^-^CD69^-^, PD1^+^ and TIGIT^+^ cells at baseline (6 days prior to infusion); 1 day pre-infusion; 1,3,7,14,28,61 and 70 days post-infusion. Red blood cells were lysed using 10x RBC Lysis Buffer (BD, cat. 555899). TILs or peripheral blood immune cells were counted using NucleoCounter NC-200 (ChemoMetec) for absolute number calculations. TILs were stained with fluorochrome-conjugated antibodies against combinations of the following antigens: CD3 (740187), CD45 (557748), CD4 (563737), CD8 (564,526 or 562,428), CD107A (562623), CD45RO(555492), CD62L (555544), PD-1 (563789), TIM-3 (565558), CD38 (562665), CD25 (564467), CD28(555730) (all from BD Biosciences); IL-7RΑ (351320), CD101 (331016) (both from Biolegend); (65–0865-14, EBIOSCIENCE). Flow cytometric data was acquired using a Cytoflex Flow cytometer (BECKMAN). FACS data was analyzed with FLOWJO Version 10.4.0 software (TREESTAR).

**Serum cytokine analysis**

Concentration of serum cytokines, including IFN-γ, TNF-α, IL-2 and IL-6, GM-CSF, IL-4, IL-5, IL-8, IL-13, and VEGF-A, was measured at baseline (6 days prior to infusion); day 1pre-infusion; 1, 3, 7, 14, 21, 28 ,61 and 70 days post-infusion by three commercially available kits (V-PLEX Proinflammatory Panel (human) 1 Kits; V-PLEX Cytokine Panel (human) 1 Kits and V-PLEX Chemokine Panel (human) 1 Kits).

**Total RNA extraction**

Total RNA was extracted from the tissues using Trizol(Invitrogen, Carlsbad, CA, USA) and RNAprep pure Cell kit DP430 (TIANGEN, Beijing, China) according to manual instruction. Subsequently, total RNA was qualified and quantified using a Nano Drop and Agilent 2100 bioanalyzer (Thermo Fisher Scientific, MA, USA).

**mRNA library construction**

Oligo(dT)-attached magnetic beads were used to purify mRNA. Purified mRNA was fragmented into small pieces, and cDNA was generated by Hieff NGS Ultima Dualmode mRNA Library Prep Kit for MGI (Yeasen, Shanghai, China) following manual instruction. The reaction product was purified by magnetic beads, followed by addition of A-Tailing Mix and RNA Index Adapters. The cDNA fragments with adapters were amplified by PCR, and then purified by Ampure XP Beads. The quality and quantity of library was assessed using the Agilent 2100 bioanalyzer, and then underwent DSN treatment. Next, the library was analyzed to ensure the high quality of the sequencing data by Agilent 2100 bioanalyzer and qPCR. The qualified library was amplified on cBot to generate the cluster on the flow cell, and the flow cell was single-end sequenced on the Illumina Novaseq 6000 platform (Tsingke-Beijing, China).

**RNA sequencing**

The sequencing data was filtered with SOAPnuke (v1.5.2) with three criteria: (1) removing reads containing sequencing adapter; (2) removing reads whose low-quality base ratio (base quality less than or equal to 5) is more than 20%; (3) removing reads whose unknown base (‘N' base) ratio is more than 5%. Then, clean reads were stored in FASTQ format and mapped to the reference genome using HISAT232(v2.0.4). Bowtie2 (v2.2.5)33 was applied to align the clean reads to the reference coding gene set and the expression level of genes was calculated by StringTie34 (v2.1.2).

**TCR-T establishment**

The α and β sequences of a sequenced TCR were linked by the 2A sequence (GSGATNFSLLKQAGDVEENPGP), and then synthesized and subcloned into the pHAGE lentiviral vector. Lentivirus was produced in 293T cells and collected according to the same protocol as before.

Cryopreserved PBMCs isolated from patient blood or Jurkat-NFAT-Luc reporter cell line were recovered and seeded in RPMI 1640 supplemented with human IL-2 (200 U/mL, Peprotech, New Jersey, USA) in a 24-well plate pre-coated with anti-CD3 and CD28 (5 μg/mL respectively, Peprotech, New Jersey, USA), at a density of 1 × 10^6^ cells per well. After 48 h, 5 × 10^5^ activated T cells were transferred to Retronectin (Takara, Japan)-coated 24-well plates and TCR virus were subsequently added, and plates were centrifuged at 2000 rpm for 50 min at 30 °C. After centrifugation, supernatants were removed and replaced with fresh medium containing 200 U/mL IL-2. Transduction efficiency was measured in 72 h. Transduced T or reporter cells were cultured with replaced fresh medium and IL-2 every 3 days, and were cryopreserved in 10 days post-transduction.

**Clinical assessment after infusion**

AEs were recorded and graded according to the Common Terminology Criteria for Adverse Events (CTCAE), version 5.0. The efficacy was evaluated according to the Response Evaluation Criteria in Solid Tumors (RECIST) v1.1.


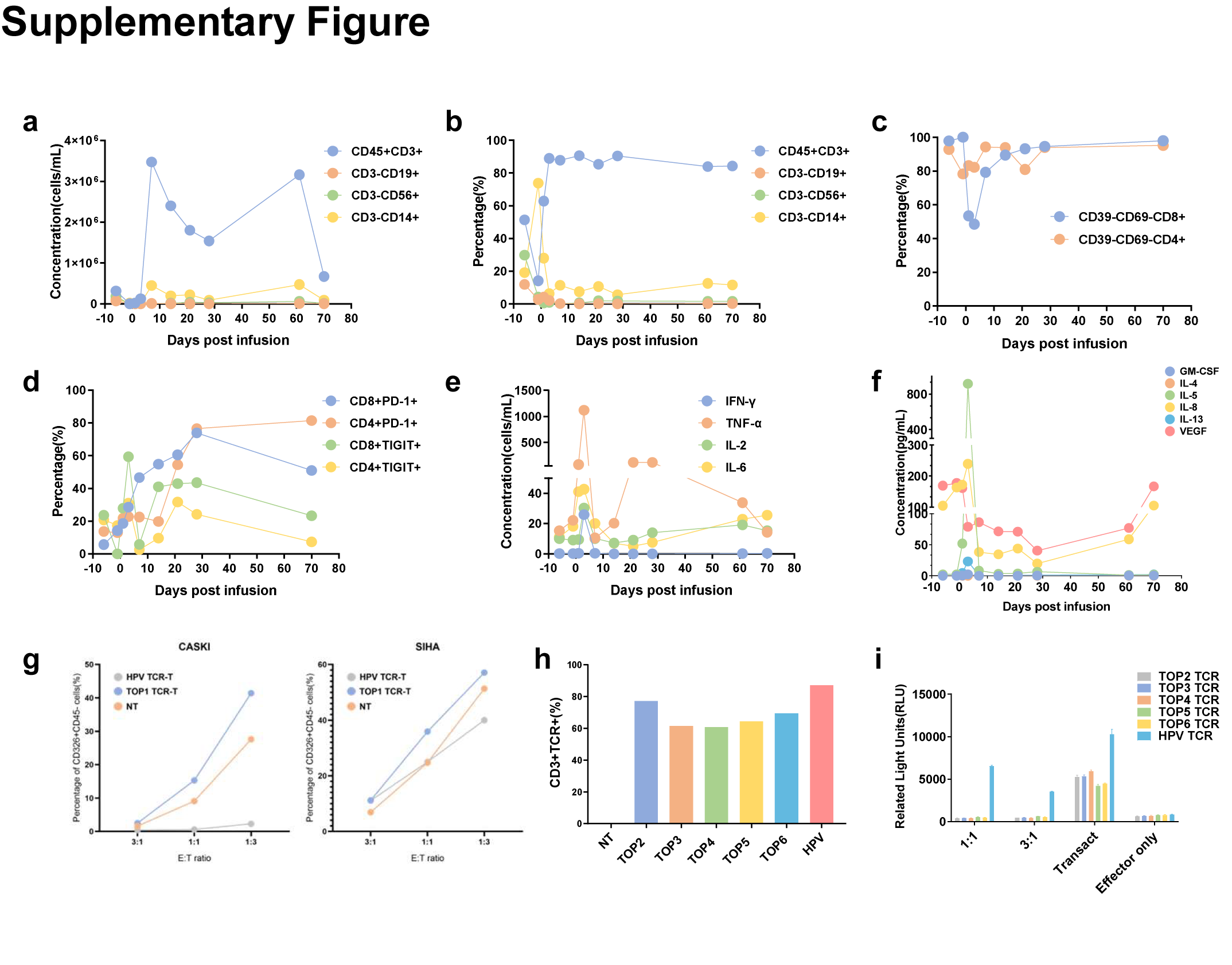


**Supplementary Figure**: Absolute counts **(a.)** and percentages **(b.)** of immune subsets in peripheral blood at indicated time points. Percentage of CD39^-^CD69^-^ **(c.)** and expression of PD-1 and TIGIT **(d.)** on CD4^+^ and CD8^+^ T cells at indicated time points. **e** and **f.** Cytokine production in serum at different sampling points. **g.** Specific killing of TOP1 TCR-T and HPV TCR-T cells against two target cell lines. **h.** Transduction efficiency of T cells from healthy donor with dominant TOP2-6 TCRs as experimental group and published HPV TCR as positive control. **i.** HPV antigens reactivity of dominant TCRs (TOP2-6) within peripheral blood derived T cells are tested via co-incubation assay with CASKI cell line.
