# Supplementary figures and images for: T cell receptor repertoire characteristics and therapeutic potential of tumor infiltrating lymphocytes (TILs) derived from metastatic lymph node in cervical cancer
Source: Mol Biomed. 2024 Nov 4;5:51. doi: 10.1186/s43556-024-00215-w (PMC11532323; doi:10.1186/s43556-024-00215-w)

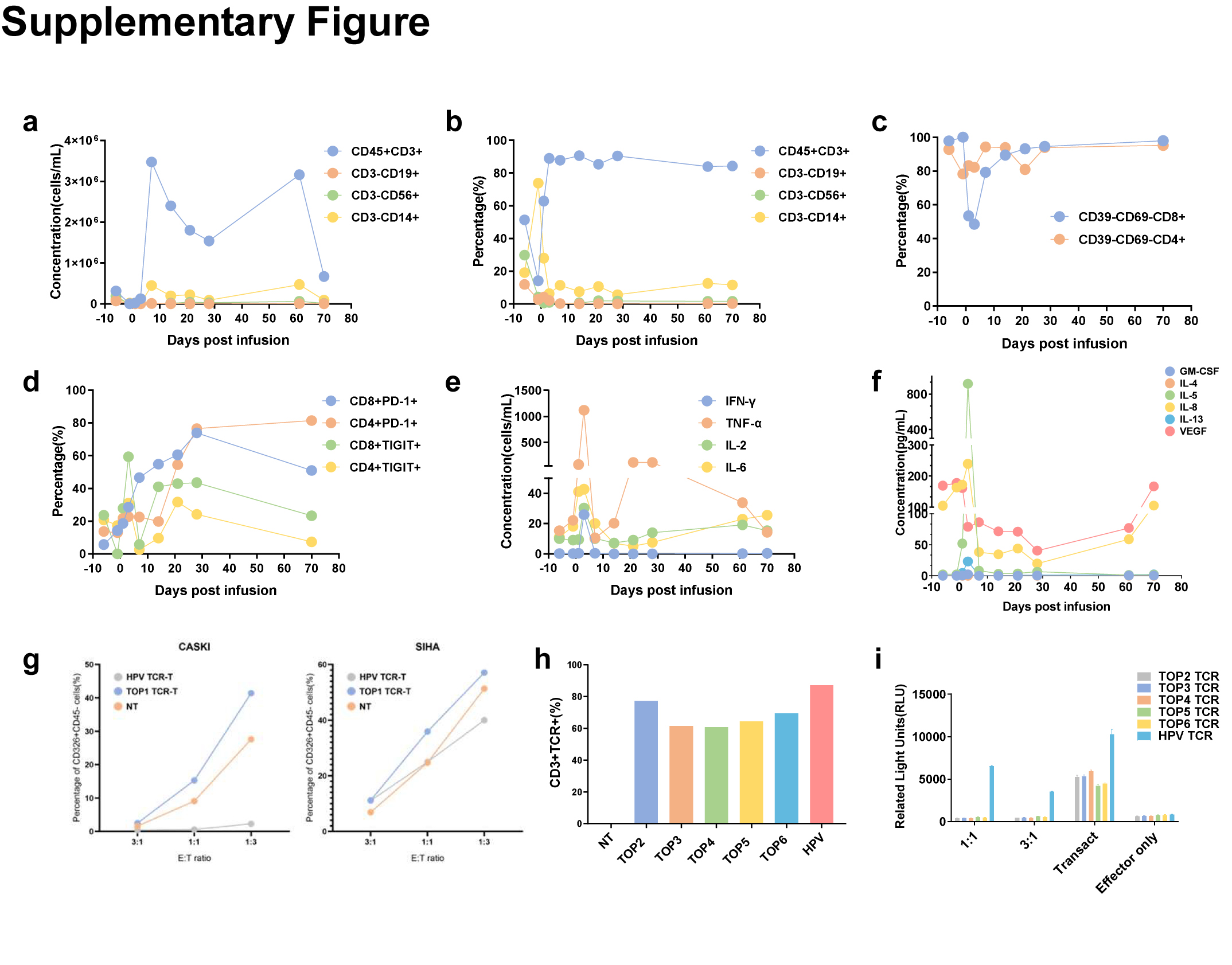

Supplement: Supplementary file 1 — Supplementary Material 1. [file 43556_2024_215_MOESM1_ESM.zip › Supplementary Figure.tif]
